# Expression and Function of TNF and IL-1 Receptors on Human Regulatory T Cells

**DOI:** 10.1371/journal.pone.0008639

**Published:** 2010-01-11

**Authors:** Frances Mercer, Lina Kozhaya, Derya Unutmaz

**Affiliations:** 1 Department of Microbiology, New York University School of Medicine, New York, New York, United States of America; 2 Department of Pathology, New York University School of Medicine, New York, New York, United States of America; University of California San Francisco, United States of America

## Abstract

Regulatory T cells (Tregs) suppress immune activation and are critical in preventing autoimmune diseases. While the ability of Tregs to inhibit proliferation of other T cells is well established, it is not yet clear whether Tregs also modulate inflammatory cytokines during an immune response. Here, we show that the expression of inflammatory cytokine receptors IL-1R1 and TNFR2 were higher on resting mature Tregs compared to naïve or memory T cells. While upon activation through the T cell receptor (TCR), expression of IL-1R1 and TNFR2 were upregulated on all T cell subsets, IL-1R1 maintained significantly higher expression on activated Tregs as compared to other T cell subsets. The decoy receptor for IL-1 (IL-1R2) was not expressed by any of the resting T cells but was rapidly upregulated and preferentially expressed upon TCR-stimulation on Tregs. In addition, we found that Tregs also expressed high levels of mRNA for IL-1 antagonist, IL-1RA. TCR-stimulation of naïve T cells in the presence of TGFβ, which induces FOXP3 expression, however did not result in upregulation of IL-1R1 or IL-1R2. In addition, ectopic expression of FOXP3 in non-Tregs, while causing significant upregulation of IL-1R1 and IL-1R2, did not achieve the levels seen in *bona fide* Tregs. We also determined that resting human Tregs expressing IL-1R1 did not have higher suppressive capacity compared to IL-1R1- Tregs, suggesting that IL-1R1 does not discriminate suppressive resting Tregs in healthy individuals. Functionally, activated human Tregs displayed a capacity to neutralize IL-1β, which suggests a physiological significance for the expression of IL-1 decoy receptor on Tregs. In conclusion, our findings that human Tregs preferentially express receptors for TNF and IL-1 suggest a potential function in sensing and dampening local inflammation.

## Introduction

Regulatory T cells (Treg) are characterized by the ability to suppress immune activation [1]. Tregs are a subset of CD4+ cells and are typically identified based on CD25 and FOXP3 expression [1]. The latter is a transcription factor also necessary for their development and function [1]. While it is well established that Tregs are highly potent in inhibiting the activation and proliferation of other T cell subsets *in vitro* and *in vivo*, the exact mechanisms of this suppression are not fully understood [2]. However, evidence from mouse models suggests that Tregs may have other anti-inflammatory properties in addition to suppressing T cell activation [1], [3]. For example, Tregs have been shown to prevent T cell independant intestinal inflammation [4]. Little is known on how Tregs mediate T cell-independent suppressive mechanisms and whether the inflammatory milieu may have modulatotry effects on the function of Tregs.

Recently, IL-1R1 and IL-1R2 were shown to be expressed on *in vitro* expanded human Tregs [5] and TNFR2 was shown to be expressed on murine and human Tregs [6]. IL-1R1 is a signaling receptor for IL-1, which mediates its function [7]. IL-1R2, instead neutralizes IL-1 either as a surface decoy receptor or in a cleaved and secreted form [7], [8], [9]. TNFR2 is an inducible receptor for TNF, that can trigger both cell survival and inflammatory signals [10].

In humans, Tregs comprise 2–5% of total CD4+ cells and similar to mouse Tregs, are crucial for proper immune function, as their absence results in massive autoimmunity [11]. The canonical murine Treg markers, FOXP3 and CD25, do not selectively define human Tregs, since these markers can be induced on other human T cells upon activation, especially in the presence of TGFβ [12], [13]. It was recently shown that IL-1R1 and IL-1R2 can be useful markers to purify Tregs from *in vitro* expanded cultures [5]. However, the expression pattern and function of these receptors on human Tregs is not yet fully characterized. Here, we show that IL-1R1 and TNFR2 are preferentially expressed on resting *ex vivo* isolated Tregs. However, upon activation both of these receptors are upregulated on other T cells subsets, although IL-1R1 maintains preferential expression on Tregs. We also found that Tregs have the capacity to neutralize IL-1β activity, suggesting that preferential expression of IL-1β decoys by these cells has a functional consequence of possibly suppressing the inflammatory cytokine milieu.

## Results

### Human Tregs preferentially express IL-1 and TNF receptors and decoys of IL-1

In order to identify new effector molecules that may contribute to Treg function, we had performed differential gene expression analysis of CD4+ cells subsets, which were isolated based on expression of CD25 and CD45RO: Naïve, (T_N_ defined as CD25-CD45RO−), Memory, (T_M_ defined as CD25−CD45RO+), Naïve Treg, (TNreg defined as CD25+CD45RO−), and Treg (CD25+CD45RO+) as described [14]. During the course of analysis of this data set we found that several cytokine receptors, IL-1R1, IL-1R2 and TNFR2, which were recently reported to be preferentially expressed on human and murine Tregs [6], [15] or *in vitro* expanded human Tregs [5] were preferentially expressed on resting or activated *ex vivo* human Tregs (data not shown). In addition to these receptors we also found that the IL-1 Receptor Antagonist (IL-1RA) was highly expressed preferentially on human Tregs (data not shown), which has not been reported before. Together, these expression profiles of pro-inflammatory cytokine receptors and their decoys prompted us to further characterize them phenotypically and functionally on human Treg subsets.

**Figure 1:**
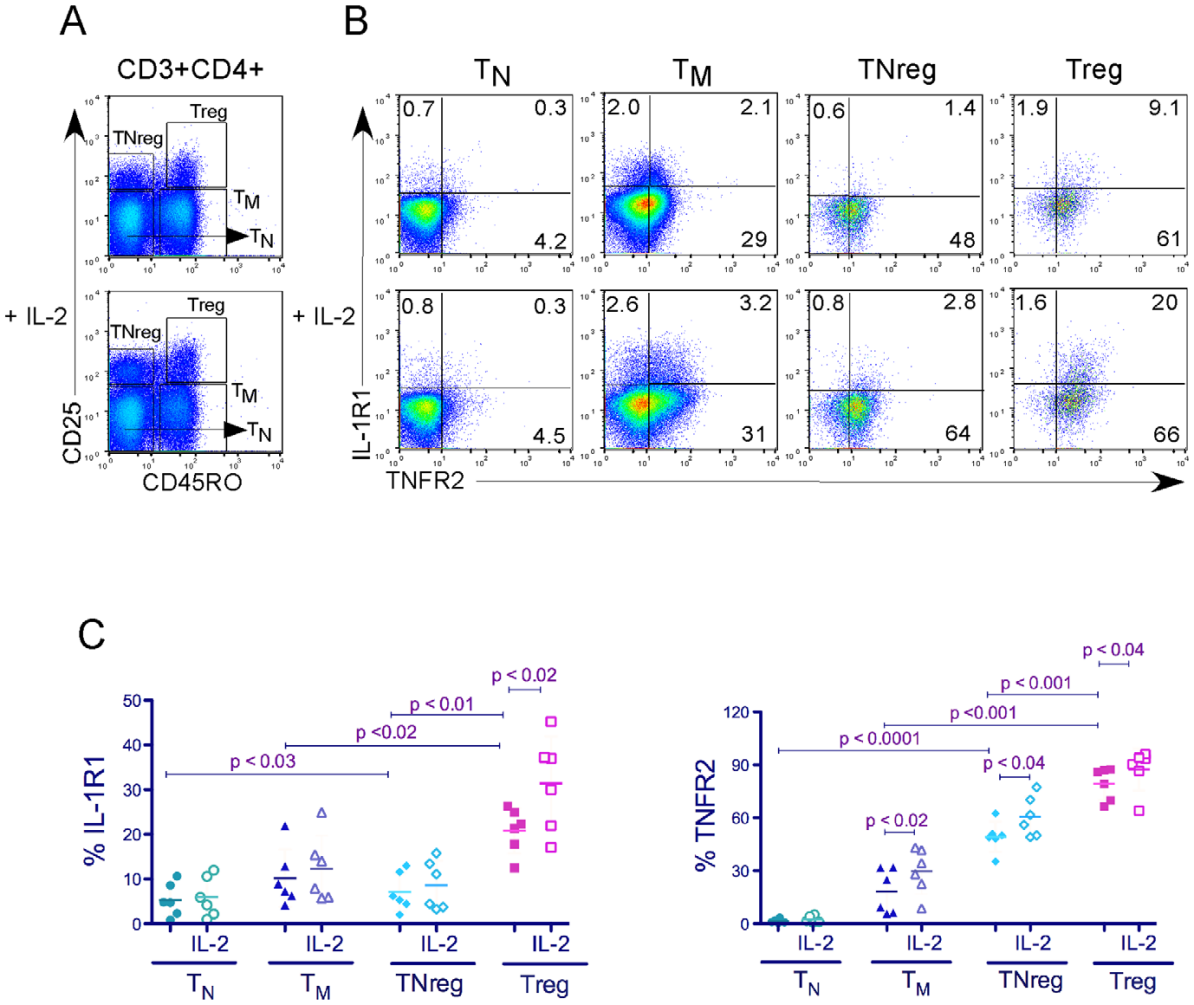
Expression of IL-1R1 and TNFR2 on human T cell subsets. (A) Total PBMC from healthy donors were stained with for CD3, CD4, CD25, CD45RO, IL-1R1 and TNFR2 after resting overnight in culture with or without IL-2. Lymphocytes were gated on CD3+CD4+, which were further gated into naïve (T_N_ CD45RO−CD25−), memory (T_M_, CD45RO+CD25−), naïve Treg (TNreg, CD45RO− CD25+), and Treg (CD45RO+CD25+). (B) Expression of IL-1R1 and TNFR2 is shown for each subset. Data is representative of 6 different donors. (C) Results calculated as in (B) are shown for 6 donors with statistical analysis.

Next, we confirmed expression of IL-1R1, IL-1R2 and TNFR2 on Tregs and other T cell subsets. PBMC isolated from blood of healthy donors were stained for CD3, CD4, CD25, and CD45RO to separate them into four subsets (Fig. 1A), which were then assessed for expression of IL-1R1 and TNFR2 using flow cytometry (Fig. 1B and S1). IL-1R1 was either absent or expressed at significantly lower levels on T_N_, TNreg and T_M_ cells compared to Tregs (Fig. 1B, 1C and Fig. S1). Tregs, and interestingly also TNregs, expressed much higher levels of TNFR2 compared to T_M_ cells, and T_N_ cells were mostly negative for TNFR2 expression (Fig. 1B, 1C and Fig. S1). The addition of IL-2 to the cultures enhanced expression of TNFR2 modestly across all subsets, and IL-1R1 specifically on Tregs (Fig. 1B, 1C and Fig. S1).

**Figure 2:**
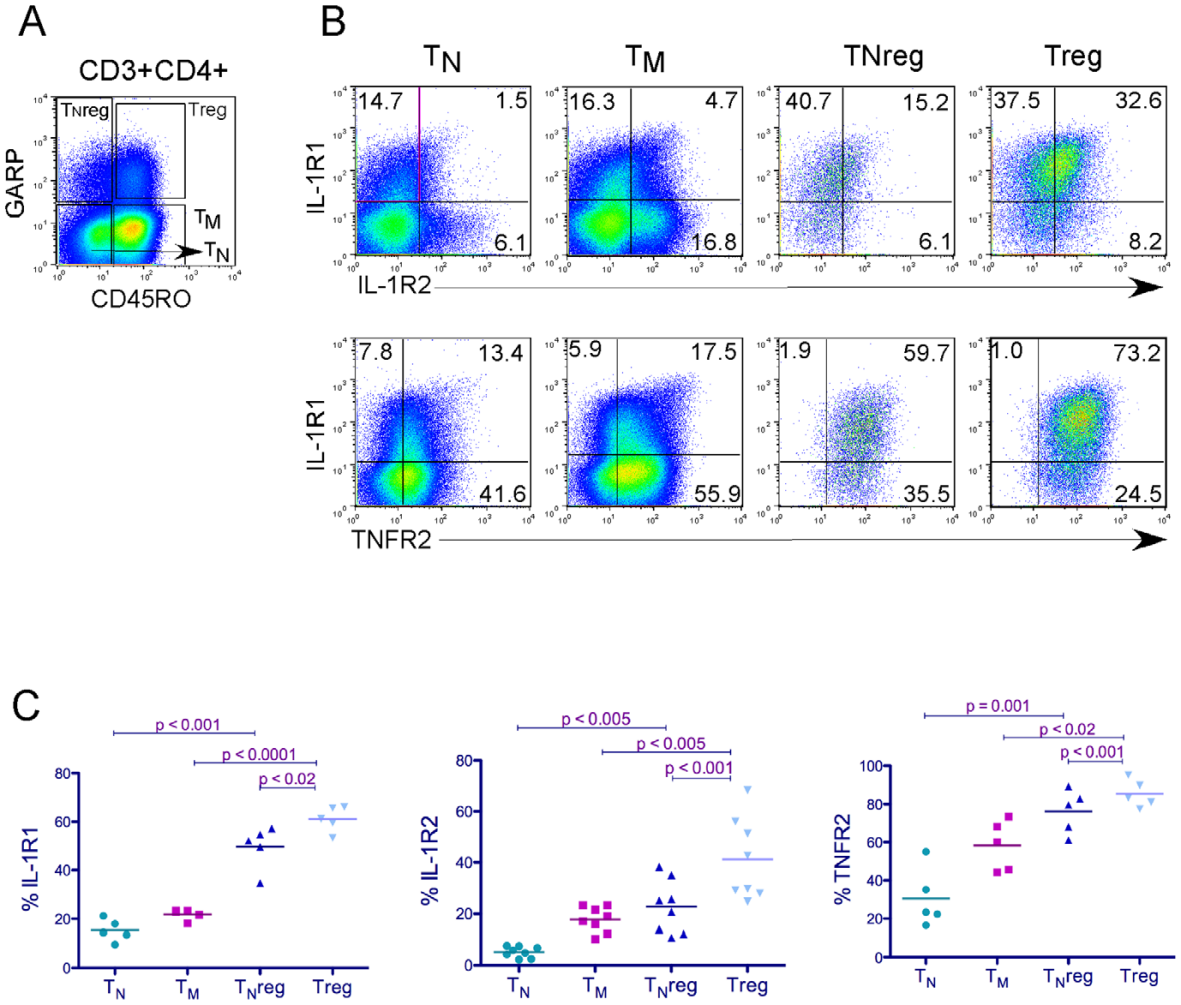
Expression of IL-1R1, TNFR2 and IL-1R2 on activated CD4+ T cell subsets. (A) Total CD4+ cells were activated with anti-CD3/CD28 coated beads for two days and stained for GARP, CD45RO, IL-1R1, IL-1R2, and TNFR2. Live cells were gated into T_N_ (CD45RO−GARP−), T_M_ (CD45RO+GARP−), TNreg (CD45RO−GARP+), and Treg (CD45RO+GARP+). (B) Expression of IL-1R2, IL-1R1 and TNFR2 are shown for each population. A representative donor is shown. (C) Statistical analysis of results shown in (B) from multiple donor blood.

In order to determine the expression of these receptors after T cell activation, we stained purified CD4+ T cells that were stimulated through the TCR. We have recently identified a cell surface molecule called GARP as a highly specific marker for activated Tregs [14], [16]. Because at 2 days post-activation, T_N_ cells still remain CD45RO negative, we used GARP and CD45RO as markers to define activated Tregs following 2 day TCR stimulation of total CD4+ cells (Fig. 2A). We then assessed expression of IL-1R1, IL-R2 and TNFR2 on these activated subsets. We found that IL-1R1 is preferentially expressed on Tregs when compared to other activated subsets (Fig. 2B, 2C and Fig. S2). TNFR2 expression increased on all subsets upon activation (Fig. 2B and 2C) but was much higher on Tregs as determined by analysis of the mean intensity of expression (Fig. S2). IL-1R2 was also modestly induced on all T cells subsets upon activation, and was highest on Tregs (Fig. 2B, 2C and Fig. S2).

We also observed high expression of IL-1RA mRNA in our microarray analysis, which we then confirmed using quantitative real time PCR in Tregs isolated from multiple donors (Fig. S3). However, we were not able to detect any protein product using intracellular cytokine staining, or multiplex cytokine assays with a 5 pg/ml limit of detection (data not shown). At this point we do not know the reason for not being able to detect IL-1RA at the protein level despite very high mRNA levels. It is plausible that this protein is secreted at very low levels only, being neutralized through IL-1R1 [17] or being rapidly degraded.

### Time-course expression of IL-1R1, IL-1R2, and TNFR2 on CD4+ subsets

**Figure 3:**
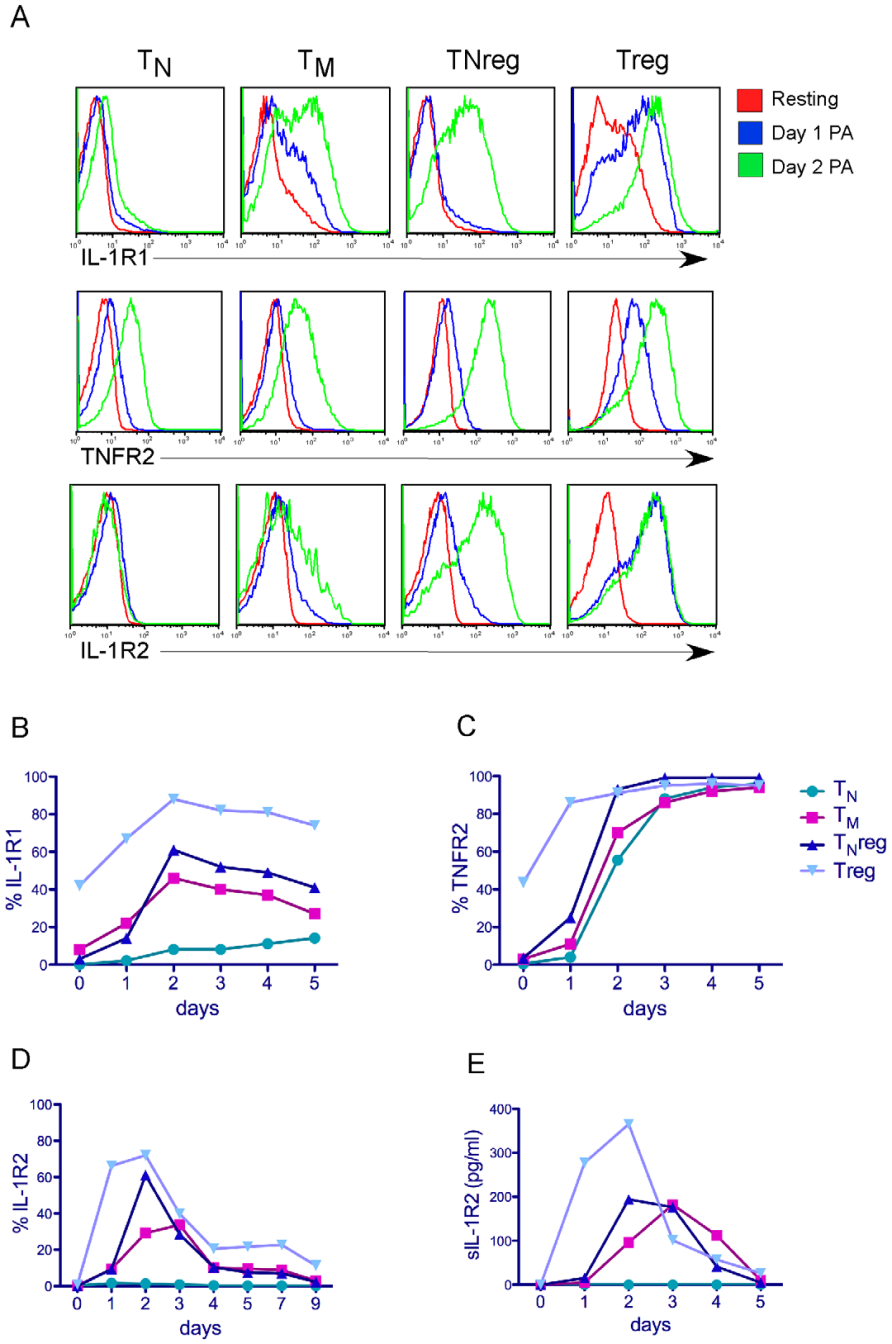
Time-course of IL-1R1, IL-1R2 and TNFR2 expression on activated CD4+ T cell subsets. (A) Total resting CD4+ cells were sorted based on CD25 and CD45RO expression into 4 subsets as described above. The subsets were then stimulated through the TCR and stained for IL-1R1, TNFR2 and IL-1R2 daily. Histogram overlays of expression on resting (red), day 1 (blue) and day 2 (green) post activation are shown. (B–D) Percentages of IL-1R1, IL-1R2 and TNFR2-expressing T cells over several days post- TCR-activation. (E) The amount of soluble IL-1R2 detected in the supernatant from T cells washed and re-plated at 0.5×10^6^/ml daily, as determined by CBA.

To determine the expression of the IL-1 and TNF receptors on sorted CD4+ subsets over-time after activation, we stimulated purified T_N_, T_M_, TNreg, and Treg cell subsets using anti-CD3/CD28 coated beads. These activated T cells were then cultured and expanded in the presence of IL-2, and stained daily for expression of IL-1R1, IL-1R2, and TNFR2. We found that IL-1R1 peaked at day 2 post-activation on T_M_ and TNreg cells (Fig. 3A), and then gradually declined over time on these subsets (Fig. 3B). Tregs, however, maintained significantly higher expression of IL-1R1 compared to other subsets even 5 days after activation (Fig. 3B). In contrast, TNFR2 was induced and maintained on nearly all T cells 2–3 days post TCR-stimulation (Fig. 3A, 3C), and therefore quickly lost its preferential expression pattern on Tregs. The expression of IL-1R2 also peaked at day 2 post-activation on T_M,_ TNreg and Treg subsets, but rapidly declined to undetectable levels for both the cell surface and secreted forms (Fig. 3D and 3E). Interestingly, T_N_ cells did not express any IL-1R2 during the time course of this activation (Fig. 3D and 3E) and the expression of IL-1R1 on these cells was also very modest even 5 days after TCR stimulation (Fig. 3B).

**Figure 4:**
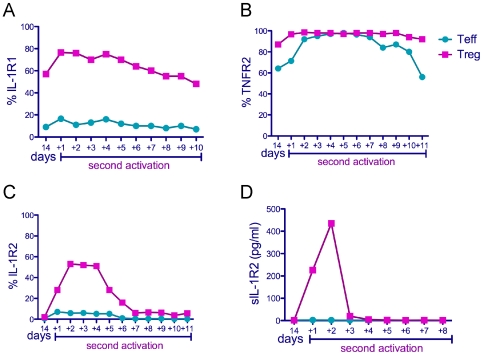
Time-course of IL-1R1, IL-1R2 and TNFR2 expression on *in vitro* expanded and reactivated T cell subsets. Sorted TNregs and T_N_ cells were reactivated at least 14 days after initial stimulation to produce expanded Treg and Teff cells, respectively. (A–C) Cells were stained at day 14 post first activation and then daily after re-stimulation through TCR (day +1, etc). (D) Supernatants were harvested from the cells restimulated washed and plated at 0.5×10^6^/ml daily and the level of soluble IL-1R2 was analyzed.

We next determined the expression pattern of the IL-1 and TNF receptors on *in vitro* expanded T cells post reactivation. We and others have shown that TNregs are precursors of canonical Tregs *in vitro* and have much greater proliferative capacity when compared to mature Tregs [18], [19]. We therefore characterized expression of IL-1R1, IL-1R2, and TNFR2 on TNreg, and T_N_ cells expanded for two weeks in culture in IL-2 containing media and then reactivated through the TCR to generate mature Treg and T effector (Teff) cells, respectively. We found that the expression of IL-1R1, and IL-1R2 followed a similar pattern in *in vitro* expanded Tregs (Fig. 4A, 4C and 4D) compared to those isolated *ex vivo* (Fig. 3B, 3D and 3E). As such, IL-1R1 continued to be expressed at higher levels on Tregs during *in vitro* expansion and was further upregulated upon reactivation (Fig. 4A). IL-1R2, on the other hand, is downregulated to background levels after two weeks of expansion but was rapidly induced upon reactivation of the Tregs (Fig. 4C and 4D). In contrast, the expression level of TNFR2 was consistently high on both Tregs and Teff cells after the *in vitro* expansion phase, albeit at slightly lower level on Teff cells, which increased to Treg levels upon reactivation (Fig. 4B).

### Regulation of IL-1R1 and IL-1R2 by FOXP3

**Figure 5:**
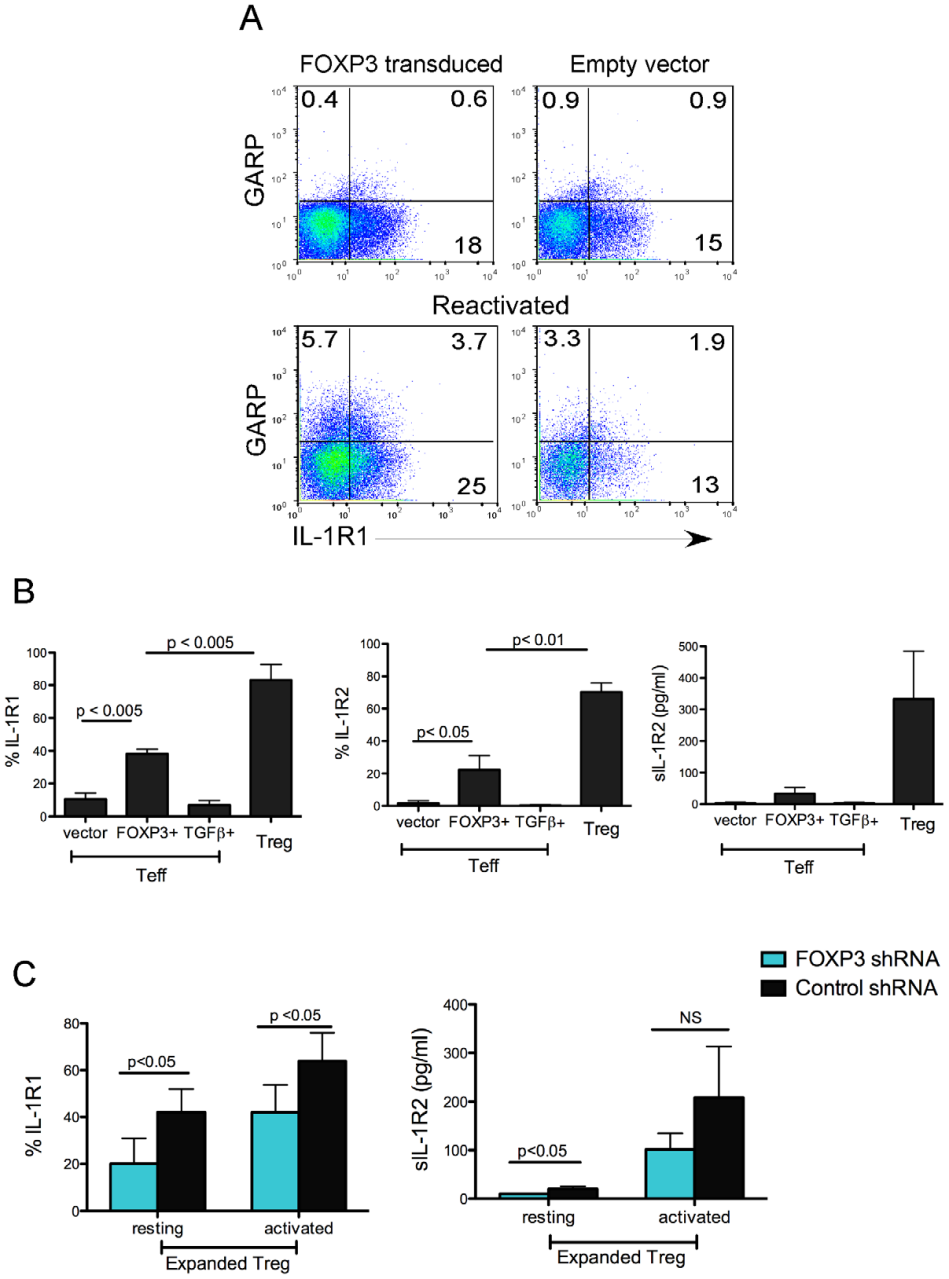
Regulation of IL-1R1 and IL-1R2 expression by FOXP3. (A) Cells ectopically overexpressing FOXP3 were generated using lentiviral transduction as described in the methods. Cells were restimulated, and stained two days later for GARP and IL-1R1 expression. (B) Expression of IL-R1, IL-1R2 and secretion of soluble IL-1R2 in empty vector transduced, FOXP3 transduced, TGFβ treated, and expanded Tregs. TGFβ treated cells were reactivated in the presence of TGFβ1 (20 ng/ml) one week after initial activation. Expanded Tregs were gated on GARP+ cells to exclude non-Tregs in the same cultures from the analysis. Data are average of three donors. (C) Comparison of IL-1R1 and soluble IL-1R2 expression on FOXP3 shRNA lentivirus treated cells. After initial infection, expansion, and sorting, cells were reactivated and stained one day later. Supernatants were analyzed for sIL-1R2 from cells plated at 0.5×10^6^/ml daily. Data are average of three donors.

The transcription factor FOXP3 is necessary, but not sufficient for the development and function of Tregs [20]. Not all FOXP3-expressing cells have suppressive capacity, as activation of T cells in the presence of TGF-β induces FOXP3 without rendering them suppressive [12], [20]. To determine whether preferential expression of IL-1 Receptors on Tregs is regulated by FOXP3 expression, we either over-expressed FOXP3 in T_N_ cells, or treated them with TGFβ to induce endogenous FOXP3. We found that ectopic expression of FOXP3 did not induce IL-1R1 on *in vitro* expanded Tregs (Fig. 5A). However, upon reactivation IL-1R1 was slightly induced on FOXP3 overepxressing cells compared to control lines (Fig. 5A), although this was still significantly less compared to *bona fide* Tregs (Fig. 5B). T_N_ cells activated in the presence of TGFβ did not upregulate IL-1R1, even after reactivation (Fig. 5B). A Similar pattern of expression was also observed on these cells for the cell surface and secreted forms of IL-1R2 (Fig. 5B).

In a reverse set of experiments, we assessed the expression of IL-1 receptors on Tregs upon silencing FOXP3 expression. We found that shRNA-mediated knockdown of FOXP3 modestly, but reproducibly downregulated IL-1R1 and IL-1R2 expression in Tregs (Fig. 5C). Collectively, these results indicate that FOXP3 makes a modest contribution to induction of IL-1R1 and IL-1R2 expression in Tregs. We did not perform similar experiments for TNFR2 expression, since this receptor is already constitutively expressed on all activated T cells.

### Can expression of IL-1R1 be used to better define resting bona fide Tregs?

**Figure 6:**
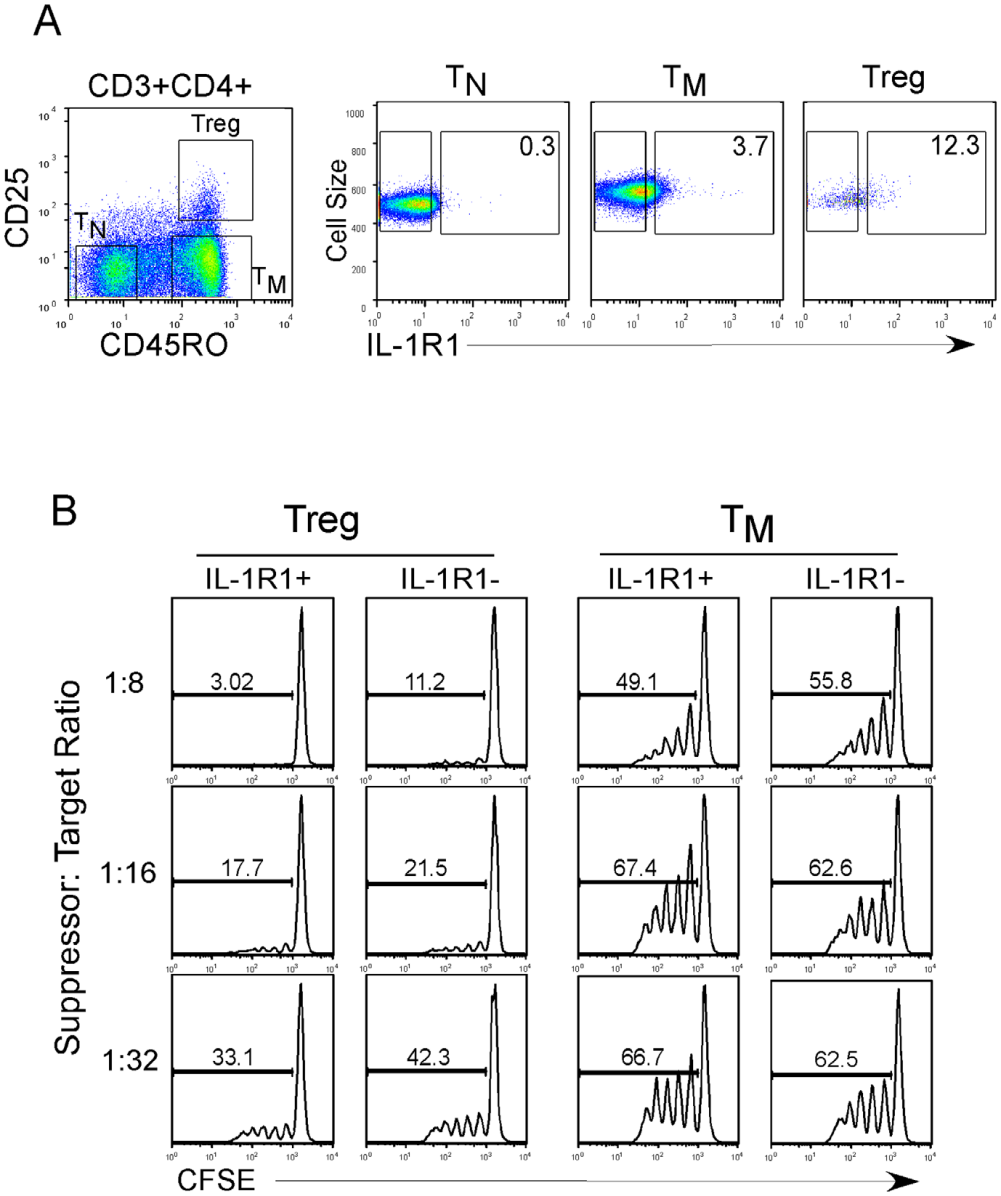
Suppressive capacity of IL-1R1 expressing Tregs. (A) Total resting CD4 cells were stained for CD25, CD45RO, and IL-1R1 expression. Cells were gated on Treg and T_M_ as described in figure 1 and further sorted based on expression of IL-1R1. Since T_N_ cells lack IL-1R1 expression, these cells were used to set the sort gate. (B) Suppression of T_N_ target cells by Tregs or Teff cells was measured for various concentrations of OKT3 and various suppressor: target ratios using the Treg suppression assay described in the methods. One representative OKT3 concentration is shown.

Numerous reports have now shown that CD25 is an imperfect marker for Tregs because it is also induced following activation of conventional T cells [12], [20]. Therefore, Treg sorts are often contaminated with recently or chronically activated cells masquerading as Tregs [16]. We have recently shown that GARP, which is specifically expressed on Tregs and TNregs upon activation, is a useful marker for isolating highly pure activated Tregs [16]. Similarly, IL-1R1 was recently used as a marker to purify activated expanded human Tregs *in vitro* [5]. Since a portion of resting Tregs express IL-1R1, we asked whether it could be useful as a marker of *ex vivo* Tregs. To address this question, we stained purified CD4+ T cells with CD25, CD45RO, and IL-1R1 antibodies and sorted Treg and T_M_ cells into subsets based on IL-1R1 expression (Fig. 6A). We then tested the suppressive capacity of these cells at multiple ratios of suppressor to target T cells. We found that there was no significant or reproducible difference in suppressive capacity between IL-1R1+ versus IL-1R1- Treg cells (Fig. 6B and Fig. S4). As expected, the IL-1R1+ or the IL-1R1- memory subset did not show any significant suppressive activity (Fig. 6B and Fig. S5). In conclusion, the expression of IL-1R1 does not discriminate suppressive Tregs *ex vivo*, at least not those isolated from healthy donors.

### Can Tregs neutralize IL-1β through expression of decoys?

**Figure 7:**
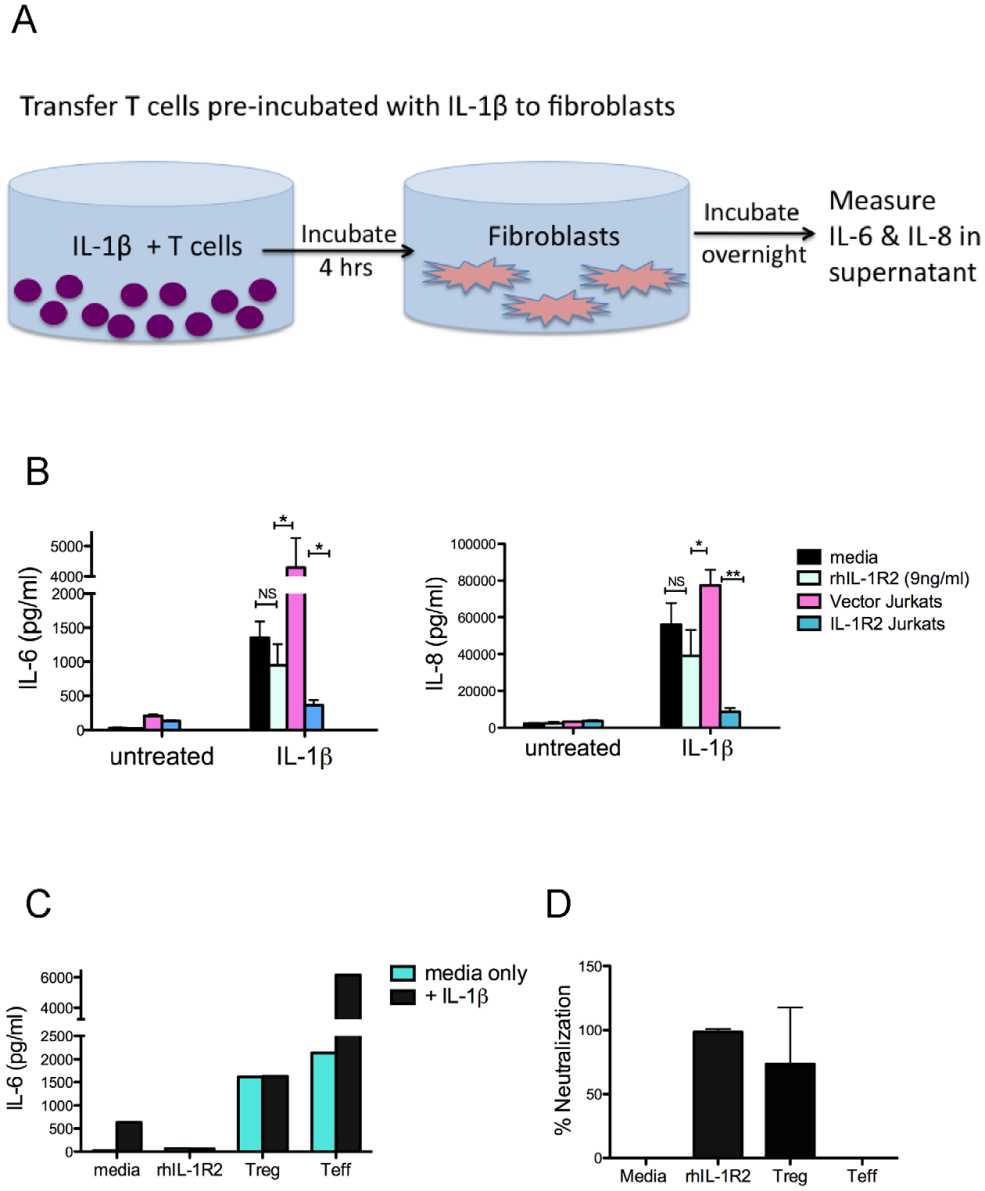
Can Tregs neutralize IL-1β activity? (A) Schematic of neutralization assay, as described in the methods. (B) Jurkat cells (10,000 cells) were cultured with recombinant IL-1β (10 pg/ml), incubated for 4 hours, and then transferred to pre-plated fibroblasts (1000 cells). Twice the amount of recombinant rhIL-1R2 (9 ng/ml) secreted by Jurkat cells overexpressing IL-1R2 was similarly preincubated with IL-1уÂand added to fibroblasts. After overnight incubation, supernatants from the fibroblasts were collected and tested for IL-6 and IL-8 using CBA. Data are average of duplicate wells and are representative of three experiments. (C) Using the same set up as described in (A), expanded Treg or Teff cells were tested for neutralization. Recombinant rhIL-1R2 (5 ug/ml) was used as a positive control for neutralization of all IL-1β. Data are representative of 3 donors. (D) Percent neutralization, calculated as described in methods, was averaged for 3 donors.

IL-1R2 is a well-characterized decoy form of the IL-1 receptor that binds IL-1 without transducing intracellular signals [21], [22], [23]. We therefore hypothesized that Tregs may directly neutralize IL-1β through their surface expression and secretion of IL-1R2. To perform this experiment we developed a highly sensitive bioassay to determine functional neutralization of IL-1β, which is outlined in Figure 7A. For this purpose we used human fibroblasts, which secrete high levels of IL-6 and IL-8 in response to low concentrations of IL-1β (Fig. 7). To determine whether T cell expression of IL-1R2 can neutralize IL-1β in this bioassay, we generated Jurkat T cells transduced to overexpress IL-1R2 (Fig. S5). We then preincubated Jurkat cells with recombinant IL-1β (10 pg/ml). After several hours of incubation, the IL-1β containing T cell mixtures were transferred to pre-plated fibroblast cultures (Fig. 7A). The secretion of IL-8 and IL-6 from the fibroblasts in response to functional IL-1β was then measured after overnight incubation (Fig. 7A). We found that Jurkat cells expressing IL-1R2 potently neutralized the effect of IL-1β compared to cells expressing vector only (Fig. 7B).

We then asked whether the potent neutralization from Jurkats overexpresing IL-1R2 is mostly due to its surface expression or the secreted form. To address this we used CBA to measure the amount of IL-1R2 secreted by IL-1R2 overexpressing Jurkats, which was 4.5 ng/ml in this assay. We found that even twice the amount of IL-1R2 found in Jurkat supernatants, when added to fibroblasts in recombinant form, did not significantly neutralize IL-1β function (Fig. 7B). Complete neutralization of IL-1β required 50 fold higher concentrations (5 ug/ml) of recombinant IL-1R2 (Fig. 7C). Thus, we concluded that much of the neutralizing activity of Jurkat cells overexpressing IL-1R2 is derived from the cell surface form, as has been previously suggested for other cell types [24].

We then assessed the capacity of Tregs to neutralize IL-1β using our bioassay. Addition of pre-activated expanded effector T cells induced high secretion of IL-6 from fibroblasts (Fig. 7C). This high background from Teff cells is expected, since they produce many inflammatory cytokines that can stimulate fibroblasts to release IL-6 or IL-8. Similarly, the addition of expanded Tregs to fibroblasts also induced a background response, albeit at lower levels compared to Teff cells (Fig. 7C). This was also not surprising because we expected some level of background from Tregs due to either contamination of non-Tregs in the culture [5] or secretion of cytokines such as IL-17 from Tregs [25], [26]. The background from these cells could also be due to secretion of low levels of IL-1β, however, we did not find any detectable levels of IL-1β (<5 pg/ml) in either Teff or Treg cultures using CBA. Nonetheless, addition of IL-1β to the cultures where Teff cells were present caused fibroblasts to further increase IL-6 secretion more than three-fold (Fig. 7C), suggesting that the background is not due to the presence of endogenous IL-1β in these cultures. In contrast to the Teff cells condition, addition of IL-1β to fibroblasts in the presence of Tregs did not result in higher IL-6 secretion by fibroblasts compared to Treg cells alone (Fig. 7C). Accordingly, we calculated that, after accounting for the background, Tregs appear to be as potent in IL-1β neutralizing capacity as recombinant IL-1β decoys (Fig. 7D). Similar neutralization effects were measured using IL-8 as a read-out in the supernatants (data not shown).

## Discussion

Numerous *in vivo* and *in vitro* studies have demonstrated the potent ability of Treg cells to suppress T cell activation and proliferation, which could account for the critical role of Tregs in preventing autoimmune diseases [1], [27]. Our findings that Tregs preferentially express the inflammatory cytokine receptors, IL-1R1, TNFR2 and IL-1R2, suggest a novel role for these regulatory cells in modulating inflammation.

IL-1β is a potent cytokine with pleiotropic functions [7], such as stimulating angiogenesis at inflamed tissue sites, triggering proinflammatory cytokine release from antigen presenting cells (APCs) and contributing to the polarization of Th17 cells [7], [28], [29]. Uncontrolled IL-1β, even at very low concentrations can cause pathology [7]. IL-1R2, the decoy form of the receptor has also been identified as deficient in human endometriosis, which is thought to be an IL-1β-induced pathology [30], [31], [32]. Therefore, the ability of Tregs to express and release IL-1R2, and possibly also IL-1RA, locally to neutralize IL-1β function could play an important role in dampening inflammation and autoimmunity.

The functional significance of expression of IL-1R1 and TNFR2 on Tregs is less clear. IL-1β is known to enhance the proliferation of conventional T cells [33], and studies in mice have also shown that IL-1 enhances expansion of FOXP3+ cells [34], thus preferential expression of IL-1R1 by Tregs could confer them with heightened sensitivity to IL-1 over other CD4+ subsets to give them a proliferative advantage. Alternatively, Tregs may express high levels of IL-1R1 simply to compete for IL-1β in the microenvironment, while remaining hyporesponsive to its effects, akin to the constitutive expression of the IL-2 receptor CD25 by Tregs, which has been proposed to deprive nearby T cells of IL-2 signals [35]. Conversely, IL-1 signals may negatively regulate or switch the phenotype of Tregs. In support of this, it was recently shown that IRAK−/− mice have higher Treg proportions compared to wild type mice, suggesting that IL-1 signaling could be negatively regulating Treg development or maintenance [36]. Similarly, IL-1β, in combination with IL-2 was recently shown to convert natural human Tregs into Th17 lineage cells [28]. Although deficiency or defects of Treg cells cause severe inflammation and autoimmunity *in vivo*, recent studies have even shown that Tregs may have pro-inflammatory properties in certain scenarios, for example, by producing IL-17 [25] or by coordinating protective response against HSV-2 infection [37].

We found that TNFR2 is also preferentially expressed on resting Tregs and is upregulated on Tregs with faster kinetics upon TCR stimulation compared to other T cell subsets. However, it is not yet clear why TNFR2 is preferentially maintained on Tregs. Since signaling through TNFR2 instead of TNFR1 generally promotes cell survival [10], this may be a mechanism for Treg maintenance at the site of inflammation through TNF signaling. TNFR2 can also function to enhance signaling through TNFR1 by increasing the concentration of TNF on the cell surface [10], [38] and is involved in costimulation of T cell activation [39].

Studies in mice have shown that TNF can enhance suppressive function and *in vitro* expansion of Tregs [6]. It was also shown that murine Tregs have the ability to neutralize TNF through shedding of TNFR [40]. In *in vitro* experiments it was also shown that soluble exogenous TNF abrogated suppression of T cell proliferation by Tregs [15]. Conversely, a recent study in malaria patients showed a correlation between serum levels of TNF, TNFR2 expression by Tregs, and enhanced Treg suppressive activity [41]. Analysis of healthy donors in the same study showed that TNFR2+ Tregs were more suppressive than their TNFR2- counterparts, suggesting that TNF signaling enhances suppressive function in human Tregs [41]. Careful delineation of the TNF response in human Tregs will be required to reveal subtle roles of TNF during autoimmune conditions and help to resolve contrasting reported findings.

Our FOXP3 silencing experiments partly implicate this transcription factor in induction of IL-1R1 and IL-1R2 expression on Tregs. Interestingly, we did not observe any significant expression of IL-1Rs on T cells stimulated with TGFβ to induce FOXP3. Even ectopic over-expression of FOXP3 at levels greater than in *bona fide* Tregs resulted only in modest expression of these receptors. A similar expression pattern is seen for GARP expression by T cells, in which ectopic over-expression of very high levels of FOXP3 could induce only very low levels of GARP [16]. Indeed, numerous reports have now confirmed that FOXP3 is not sufficient for *bona fide* human Treg cell differentiation, and that other transcription factor(s) could be required for full Treg cell programming [20], [42]. Alternatively, Tregs may have undergone chromatin changes during differentiation that better allow FOXP3 either directly or indirectly to turn on target genes [43]. Full expression of GARP and IL-1Rs in T cells may therefore require additional transcription factors or the *bona fide* Treg chromatin pattern.

Currently, identification of resting human Treg cells relies on expression of high levels of CD25 and expression of FOXP3. While these markers identify Tregs reliably in healthy individuals, chronic immune activation in diseases such as HIV infection can confound identification of Tregs due to an increase in effector T cells with a similar phenotype [16]. We have recently identified the cell surface molecule GARP as a highly specific marker for isolating recently activated Tregs, however GARP is not expressed on any resting Tregs [16], and thus is only useful in defining Tregs after activation. Recently, Tran et al. showed that IL-1R1, among other markers, could be utilized to purify expanded Treg populations *in vitro*, which are normally contaminated with overgrowth from non-Treg cells present in initial CD25+ sorts [5]. Our results that expanded Tregs express higher levels of IL-1R1 compared to expanded effector T cells complement these findings. However, our experiments with resting *ex vivo* derived Tregs suggest that IL-1R1+CD25+ T cells are not significantly enriched with suppressive Tregs compared to IL-1R1-CD25+ T cells. We conclude that IL-1R1 expression does not affect Treg ability to suppress T cell proliferation when isolated from healthy individuals, but more likely represents a specialized subset of Tregs that are more equipped to respond to IL-1 at the site of inflammation. It is possible, however, that IL-1R1 could be a Treg-specific marker in chronic activation disease conditions such as HIV infection, in which higher levels of CD25+ cells that are not suppressive are present, possibly due to chronic immune activation [16], [44].

In summary, our findings reveal that Tregs, besides controlling T cell activation, may have an additional function of suppressing pro-inflammatory cytokines. In addition, the expression of TNF and IL-1 receptors on Tregs may have important functions in modulating their differentiation or homeostasis during chronic immune activation or inflammation.

## Materials and Methods

### Cell purification and culture

Blood samples were obtained from anonymous healthy donors as buffy coats (New York Blood Center). New York Blood Center obtains written informed consent from all participants involved in the study. Because all the samples were sent as anonymous, Institutional Review Board at New York University medical center determined that our study was exempt from further ethics approval requirement. Peripheral Blood Mononuclear cells (PBMC) were isolated with Ficoll-Hypaque (Amersham Pharmacia). CD4+ T cells were isolated from PBMC using magnetic bead sorting (Invitrogen, Dynabeads) and monocyte derived dendritic cells (DC) were generated from CD14+ cells as previously described [45]. Purified cells were cultured in RPMI (Life Technologies, Carlsbad, CA) media containing fetal calf serum (FCS) (Atlanta Biologicals, Lawrenceville, GA) as previously described [45]. To activate cells for expansion in vitro and in experiments other than suppression assays, anti-CD3 and anti-CD28 coated beads (Dynabeads, invitrogen) were used at a bead: cell ratio of 1∶4 in media containing IL-2 [45].

### Real-time PCR analysis

Total RNA was isolated from flash-frozen cells using Qiagen RNeasy© mini kit, and cDNA generated using High capacity reverse transcriptase kit (Applied Biosystems). Taqman primer/probe mixtures were purchased from Applied Biosystems: IL-1RA (Hs00277299_m1) β-Actin (Hs99999903_ml). Samples were run on applied Biosystems 7300 apparatus. Data were normalized to β-Actin for each sample.

### Staining with antibodies and FACS analysis

Cells were stained in complete RPMI media or PBS+2% FCS and 0.1% sodium azide for 30 minutes at 4°C and washed before running on BD LSR-II flow cytometer. Cell sorting was done using BD FACS Aria (BD Biosciences). Data was analyzed using FlowJo software (Treestar) and gated on live cells based on forward and side scatter properties. In samples of recently activated cells, non-specific background from the beads was gated out in an irrelevant channel. The following antibodies were used in the stainings and sortings: CD25-PE, CD25-APC, CD45RO-FITC (BD Pharmingen) and CD-45RO-Pacific Blue (Biolegend), IL-1R1-PE (R&D systems FAB226F), TNFR2-Fluorescein (R&D systems FAB226F), FoxP3-APC (Biolegend), IL-1R2-biotin (BD pharmingen 552402), GARP pure (Alexis Biochemicals), and secondary antibodies; streptavidin-APC (eBioscience), anti-mIgG2b biotin (BD Pharmingen), and streptavidin APC (BD Pharmingen). For samples co-stained with GARP and IL-1R2, a directly conjugated secondary anti-mouse IgG (BD Pharmingen) was used after GARP instead of the biotinylated secondary antibody.

### Cytokine measurements in supernatants

Cytokine Bead Assay (CBA) flex sets were purchased from BD bioscience for human IL-1R2, IL-1β, IL-6, and IL-8. Experiments were preformed according to the manufacturer's instructions and samples were run on BD LSR-II flow cytometer. Geometric mean intensities for each bead were normalized to a standard recombinant protein curve for each cytokine.

### Treg suppression assay

Resting Naïve CD4+ cells (targets) were labeled with CFSE and plated at 3×10^4^/well in 96-well u-bottom plates. Suppressor cells were added at various ratios, and cells were activated using DC (generated as described above) at a DC: target cell ratio of 1∶10 and anti-CD3 antibody (ATCC clone OKT-3) at 10–100 ng/ml. All cells were washed at least twice in complete media to remove any cytokines. CFSE dilution was measured on BD LSRII flow cytometer 4 and 5 days later. Percent suppression was calculated as previously described [18].

### Ectopic gene expression and shRNA mediated gene silencing

IL-1R2 cDNA was purchased from Origene technologies and subcloned into a lentiviral vector. FOXP3 was cloned as previously described [45], and FOXP3 antisense oligos were generated as previously described [14]. Lentiviruses expressing IL-1R2, FOXP3 or a FOXP3 shRNA oligo were generated as previously described [45]. Freshly sorted T_N_ or TNreg CD4+ cells were activated with anti-CD3/CD28 beads at a bead: cell ratio of 1∶4, and transduced with a multiplicity of infection of 3. Cells were expanded in IL-2 containing media for 2 weeks and sorted for their GFP or RFP marker genes using BD FACS Aria before reactivation. Sorted cells were used to measure soluble protein levels whereas unsorted cells were used for stainings.

### Neutralization assay

Human bone marrow fibroblasts (obtained from NDRI) expanded for 18 days, were plated in 6 well plates at 15×10^4^/well and rested in RPMI for at least one day. Cells were trypsinized and re-plated in 96-well flat bottom plates at 10^3^ cells/well for at least 4 hours and media was aspirated before addition of T cells and IL-1β. Supernatants were taken and frozen 8–12 hours later and analyzed as described above. Percent neutralization was calculated as [amount of IL-6 induced from recombinant IL-1β alone]–[amount of IL-6 induced in the presence of T cell from recombinant IL-β alone] divided by [amount of IL-6 induced from recombinant IL-1β alone]. Recombinant IL-1β and sIL-1R2 were purchased from R&D systems. Recombinant IL-6 and IL-8 used to generate standard curves were provided with the BD CBA flex set kit.

### Statistical analysis

Receptor expression statistics in total PBMC, CD4+ cells, and FOXP3 overexpressing cells were done using a paired, two tailed t-test. Analysis of FOXP3 knockdown cells was done using a paired one-tailed t test. Suppression Assay statistics were done using a two-tailed paired t test. All statistics were done using GraphPad Prism software.

## Supporting Information

Figure S1 Expression of IL-1R1 and TNFR2 on resting CD4+ subsets. Histogram overlays of isotype controls (red) and specific antibody (blue) with the percent of positive cells, shown for IL-1R1 and TNFR2 expression in CD4+ subsets analyzed from freshly isolated total PBMC. Geometric means of fluorescence intensity are shown in parentheses.
(0.60 MB TIF)

Figure S2 Expression of IL-1R1, TNFR2 and IL-1R2 on activated CD4+ subsets. Histogram overlays of isotype controls (for IL-1R1 and TNFR2 stainings) or secondary only (for IL-1R2) staining. Geometric means of intensity are shown in parentheses.
(0.64 MB TIF)

Figure S3 Expression of IL-1RA mRNA in CD4+ subsets. mRNA levels of IL-1RA are shown for different CD4+ subsets. T cells were sorted as described above and activated with anti-CD3/anti-CD28 coated beads overnight. cDNA was prepared as described in methods, and real time PCR analysis for IL-1RA was done. Data are normalized to β-Actin levels and are shown as fold expression over the lowest expressing subset.
(0.35 MB TIF)

Figure S4 Suppression of T cell activation by IL-1R1+ or IL-1R1- Tregs. Graphical representation of percent suppression of data in figure 6B. Statistical analysis was performed using different suppressor: target ratios.
(0.59 MB TIF)

Figure S5 Ectopic expression of IL-1R2 on Jurkat cells. FACS histogram overlay of IL-1R2 expression in Jurkat cells were transduced with IL-1R2 encoding lentivirus (blue) or empty vector control (red). Both viruses encode GFP as amarker, thus cells were stained for IL-1R2 and gated on GFP+ cells.
(0.31 MB TIF)
