# Supplementary figures and images for: Expression and Function of TNF and IL-1 Receptors on Human Regulatory T Cells
Source: PLoS One. 2010 Jan 11;5(1):e8639. doi: 10.1371/journal.pone.0008639 (PMC2799662; doi:10.1371/journal.pone.0008639)

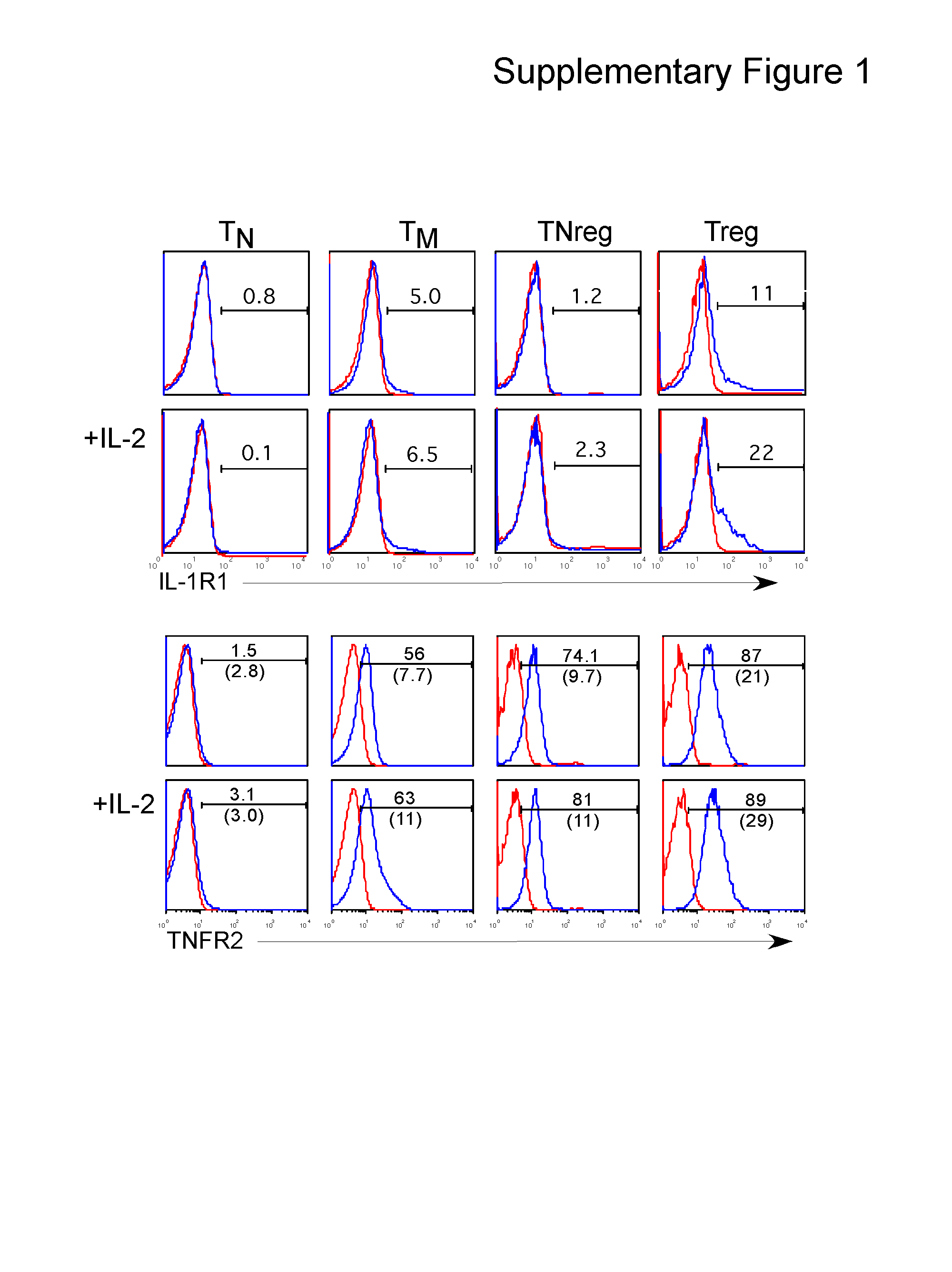

Supplement: Figure S1 — Expression of IL-1R1 and TNFR2 on resting CD4+ subsets. Histogram overlays of isotype controls (red) and specific antibody (blue) with the percent of positive cells, shown for IL-1R1 and TNFR2 expression in CD4+ subsets analyzed from freshly isolated total PBMC. Geometric means of fluorescence intensity are shown in parentheses. (0.60 MB TIF) [file pone.0008639.s001.tif]

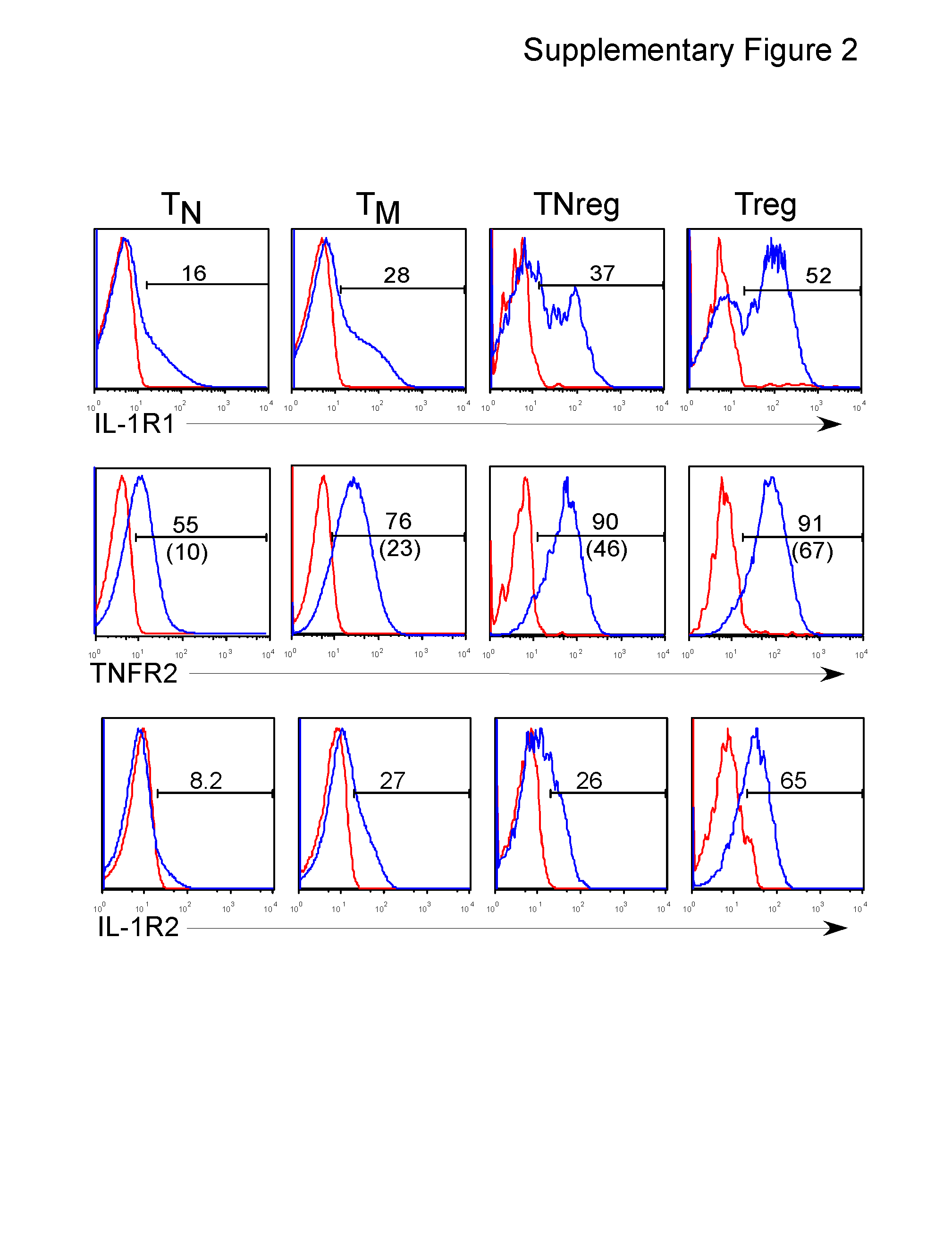

Supplement: Figure S2 — Expression of IL-1R1, TNFR2 and IL-1R2 on activated CD4+ subsets. Histogram overlays of isotype controls (for IL-1R1 and TNFR2 stainings) or secondary only (for IL-1R2) staining. Geometric means of intensity are shown in parentheses. (0.64 MB TIF) [file pone.0008639.s002.tif]

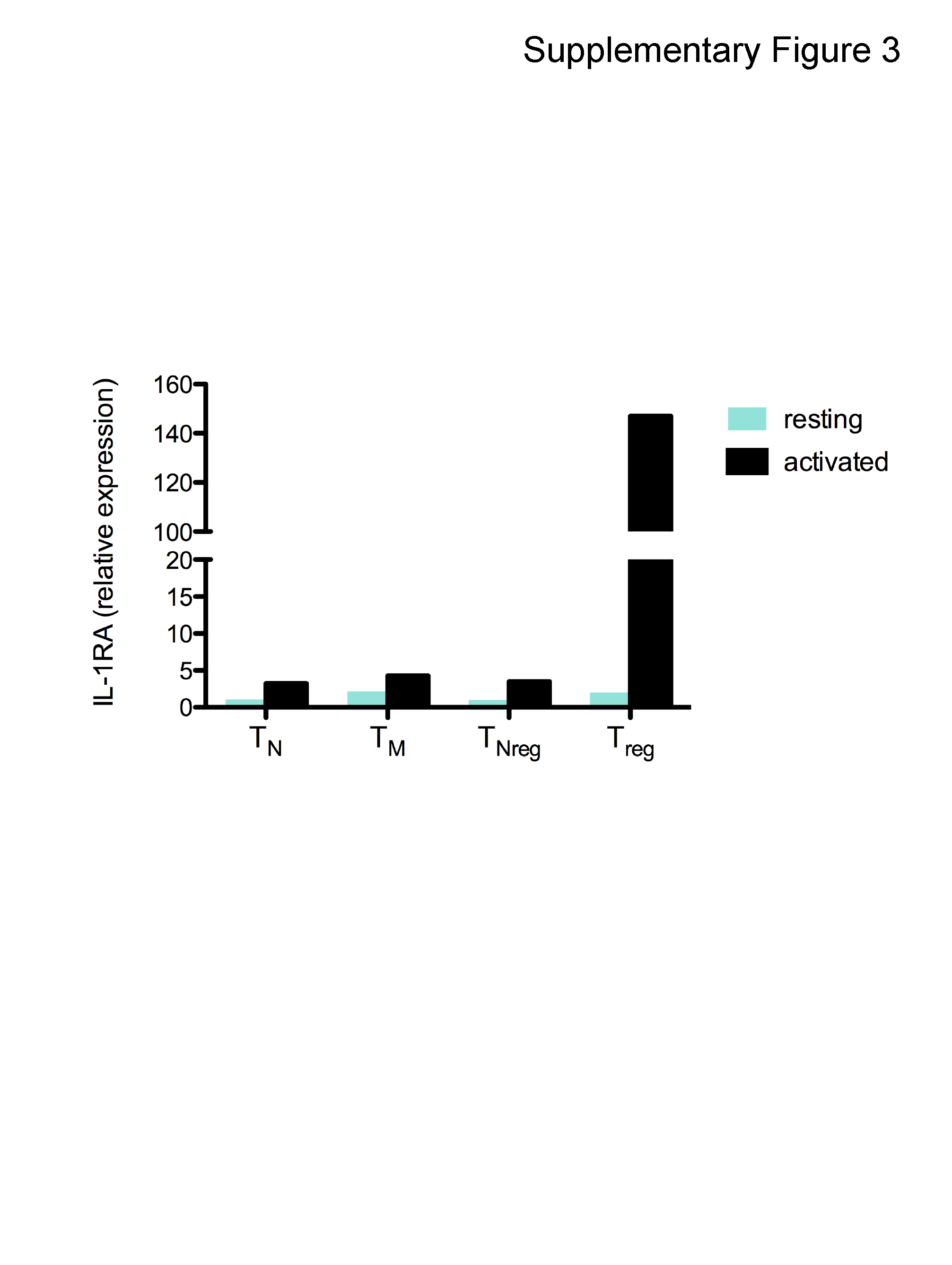

Supplement: Figure S3 — Expression of IL-1RA mRNA in CD4+ subsets. mRNA levels of IL-1RA are shown for different CD4+ subsets. T cells were sorted as described above and activated with anti-CD3/anti-CD28 coated beads overnight. cDNA was prepared as described in methods, and real time PCR analysis for IL-1RA was done. Data are normalized to β-Actin levels and are shown as fold expression over the lowest expressing subset. (0.35 MB TIF) [file pone.0008639.s003.tif]

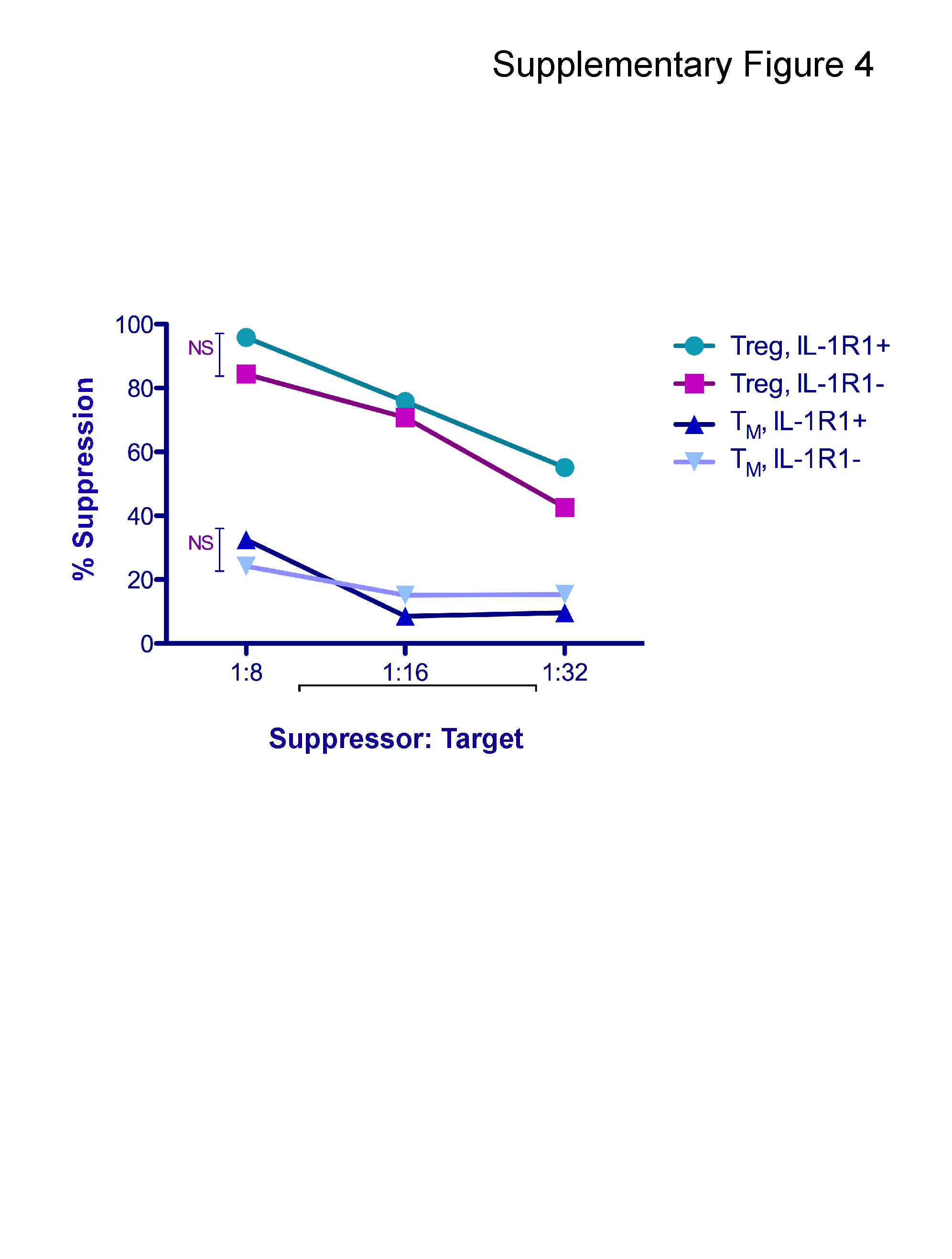

Supplement: Figure S4 — Suppression of T cell activation by IL-1R1+ or IL-1R1- Tregs. Graphical representation of percent suppression of data in figure 6B. Statistical analysis was performed using different suppressor: target ratios. (0.59 MB TIF) [file pone.0008639.s004.tif]

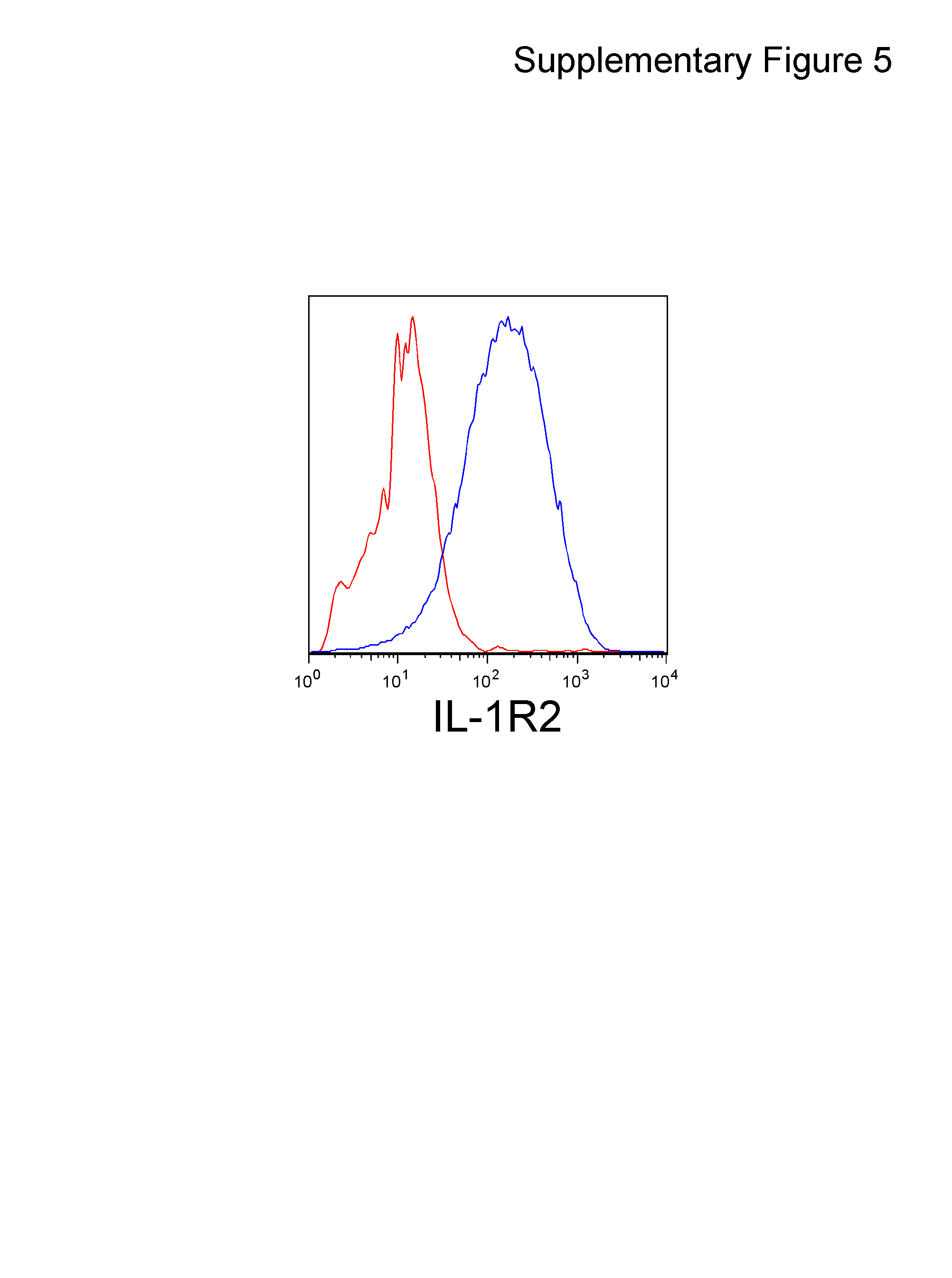

Supplement: Figure S5 — Ectopic expression of IL-1R2 on Jurkat cells. FACS histogram overlay of IL-1R2 expression in Jurkat cells were transduced with IL-1R2 encoding lentivirus (blue) or empty vector control (red). Both viruses encode GFP as amarker, thus cells were stained for IL-1R2 and gated on GFP+ cells. (0.31 MB TIF) [file pone.0008639.s005.tif]
